# Intraoperative Radiotherapy in the Treatment of Breast Cancer: A Review of the Evidence

**DOI:** 10.4061/2011/375170

**Published:** 2011-06-01

**Authors:** Norman R. Williams, Katharine H. Pigott, Mohammed R. S. Keshtgar

**Affiliations:** ^1^Clinical Trials Group, Division of Surgery and Interventional Science, University College London (UCL) Medical School, Archway Campus, London, N19 5LW, UK; ^2^Radiotherapy Department, Royal Free Hospital, London NW3 2QG, UK; ^3^Department of Surgery, Royal Free Hospital, London NW3 2QG, UK

## Abstract

The surgical treatment of early breast cancer has evolved from the removal of the entire breast and surrounding tissues (mastectomy) to the removal of the tumour together with a margin of healthy tissue (lumpectomy). Adjuvant radiotherapy, however, is still mainly given to the whole breast. Furthermore, external beam radiotherapy is often given several months after initial surgery and requires the patient to attend the radiotherapy centre daily for several weeks. A single fraction of radiotherapy given during surgery directly to the tumour bed (intraoperative radiotherapy) avoids these problems. The rationale and level-1 evidence for the safety and efficacy of the technique are reviewed.

In 1867, a surgeon at the Middlesex Hospital in London published a paper providing evidence for the local origin of breast cancer; after partial removal of the breast, recurrences were generally near the scar [1]. He thought that the recurrences spread centrifugally from the focus through the lymphatics, and the best treatment was to remove as much breast and surrounding tissue as possible. In 1894, Halsted published the results of a radical mastectomy in 50 patients—a local recurrence rate of 6%, which was extremely low by the standards of the time [2]. The technique was adopted enthusiastically and developed further with the extended radical mastectomy (sometimes together with amputation of the upper arm), culminating in the super-radical mastectomy [3].

Coincidentally, Wilhelm Röntgen discovered X-rays in 1895 and the Curies discovered radium in 1898; soon after, radiation was used to treat breast cancer, with variable results [4]. However, surgical removal of the whole breast remained the treatment of choice until the 1970s when results from trials comparing mastectomy with a combination of breast-conserving surgery and whole-breast radiotherapy showed that they were equally effective in terms of survival and considerably less traumatic for the patient [5]. In addition, radiotherapy reduced the risk of local recurrence by 75%, which resulted in a disease-free survival advantage [6] and indeed overall survival [7]. 

Currently, postoperative radiotherapy to the whole breast with a boost to the tumour bed is regarded as an adjuvant treatment to breast-conserving surgery. However, if the surgical component of the therapy has moved from whole breast (mastectomy) to local (lumpectomy), why cannot the same logic be applied to radiotherapy particularly in this era of mammography where screen-detected lesions are very small?

It is interesting to note that pioneering work on local treatments was performed by Geoffrey Keynes, a surgeon at St Bartholomew's Hospital in London. In 1922, he experimented with the use of radium encased in hollow platinum needles that were inserted around the tumour and lymphatics. Keynes combined this local radiotherapy with local excision (lumpectomy) [8]. Unfortunately, radium was difficult to obtain and handle, so the technique never caught on.

The results of many observational studies and clinical trials have demonstrated that around 90% of recurrent disease in the breast after breast conserving surgery is within the index quadrant, whether or not whole-breast external beam radiotherapy has been given, see Table 1. Furthermore, after adjuvant endocrine therapy, the chance of a local recurrence outside the index quadrant is no more than the risk of a new contralateral tumour [9].

There is evidence (described below) that ipsilateral breast cancer recurrence is in fact two distinct diseases, namely: true recurrence where the cancer is not completely removed and remaining cells grow to form a recurrence; and a second primary cancer, a tumour arising independently of the index tumour. This distinction is important as localised therapy should be judged on its ability to reduce true recurrences, but it will not be expected to have an effect on new primary cancers.

In a retrospective review of 397 patients with ipsilateral breast tumour recurrence, Yi et al. [10] reported that about half were classified as new primary cancers by two methods of assessment using data such as tumour location, histologic subtype, and hormone receptor status. Patients classified as having new primary cancers had better outcomes than those with true local recurrences, specifically 10-year overall and disease-specific survival rates and likelihood of developing subsequent metastatic disease. However, they were more likely to have a contralateral breast cancer.

Komoike et al. [11] described a cohort of 161 Japanese patients with ipsilateral breast tumour recurrences and classified them as either true recurrences or new primary cancers, based on tumour location and pathological findings. The true recurrences were associated with a high rate of lymph node metastases and a shorter disease-free interval than new primary cancers.

The records of 130 patients with ipsilateral breast tumour recurrence were reviewed by Smith et al. [12] and classified as true recurrence if located in the same position from the primary tumour bed, was of the same histologic subtype, or had the same DNA flow cytometry (remained aneuploid). Patients with new primary cancers were found to have better overall, distant disease-free, and cause-specific survival.

So, it is entirely possible that dormant cancers in the breast, distant the primary tumour, do not need any intervention. In other words, not all cancers will grow to become a clinical problem in an individual's lifetime, a situation analogous to prostate cancer, where many men die with, but not of, prostate cancer; men with nonlethal disease do not benefit from treatment [13]. The evidence to support the notion of latency amongst microscopic foci of breast cancer has been well documented [14, 15].

Whole-breast radiotherapy is not without risk. Treatment regimens have become safer since the identification of long-term side effects such as increased mortality from myocardial infarction after radiotherapy for left-sided breast cancer [16]. But, no matter how carefully applied, healthy tissues such as the heart, ribs, and lungs do receive a dose of radiation. 

Other issues with whole-breast radiotherapy include the following.

- The fractionated doses take between 3 and 7 weeks to deliver, which is a great inconvenience for women who work or look after grandchildren or ill adults or for the elderly who find the daily journeys exhausting. Women in the developing world or those in wealthy countries who live in rural areas more than 100 miles from a centre are denied the chance of breast-conserving surgery and must have a mastectomy, or else are at great hazard of local recurrence if the treatment is omitted [17, 18].
- The radiotherapy equipment is expensive to purchase and to run and requires installation in a shielded building. In the UK, the treatment of breast cancer accounts for approximately a third of the workload of radiotherapy departments.
- Geographic misses are commonplace in postoperative attempts to target the tumour bed [19].
- Cosmesis is often impaired by the short- or long-term radiotoxicity.
- Delays in the start of chemotherapy or delays in the start of radiotherapy in order to accommodate chemotherapy might compromise either modality [20]. A delay of over six weeks has been shown to significantly increase the risk of recurrence at 5 years [21].

Risks associated with accelerated partial breast irradiation include persistent seroma, postprocedural infection, erythema, telangiectasia, edema, blistering, skin thickening, fibrosis, tenderness, and pain; in general, these toxicities are modest and acceptable [22, 23].

Clinicians are increasingly adopting the view that perhaps it is not necessary to irradiate the whole breast, but rather to restrict treatment to the immediate area around the tumour bed or index quadrant. Accelerated partial breast irradiation (APBI) aims to decrease the volume of breast treated and increase the daily fraction size of radiation so that the entire dose can be delivered within 1 week (instead of 3–7 weeks). Techniques include linac-based intensity-modulated radiotherapy, multicatheter interstitial brachytherapy, balloon-based APBI using the MammoSite brachytherapy applicator (Hologic, Inc., Mass, USA), and a newly developed, modified form of balloon-based brachytherapy called Xoft Axxent Electronic Brachytherapy (Xoft, Inc., Calif, USA). A review of randomised trials and prospective single-arm studies led the American Society for Therapeutic Radiation Oncology to issue a consensus statement regarding groups of patients who could be treated by APBI, while urging that further research was required [24].

There are currently seven ongoing randomized trials testing various methods of APBI against whole-breast radiotherapy [25]. However, they are not comparable since they vary in inclusion criteria, total dose, fractionation, volume, and timing related to chemotherapy and hormone treatment. The National Surgical Adjuvant Breast and Bowel Project and the Radiation Therapy Oncology Group have noted this shortcoming which is being addressed in an intergroup study randomising patients with early-stage breast cancer to whole-breast irradiation versus APBI (using either interstitial brachytherapy, Mammosite balloon catheter, or 3D conformal external beam); accrual is expected to be completed soon (NSABP B-39/RTOG 0413, [26]).

There is, however, one type of APBI that has already generated level-1 evidence—intraoperative radiotherapy (IORT) given as a single fraction to the tumour bed during surgery.

The technique of IORT using INTRABEAM (Carl Zeiss Surgical, Oberkochen, Germany), termed TARGIT, was pioneered in London [27] and allows the patient to receive a single fraction of radiotherapy as soon as the primary tumour is excised, during the same anaesthetic. Advantages of this approach include delivering the radiation immediately, ensuring the radiation is delivered to the tumour bed under direct vision, thus avoiding a “geographical miss”, and decreasing costs to the healthcare providers.

INTRABEAM is a mobile, miniature X-ray generator powered by a 12-volt supply. Accelerated electrons strike a gold target at the tip of a 10 cm long drift tube with a diameter of 3 mm, resulting in the emission of low-energy X-rays (50 kV) in an isotropic dose distribution around the tip. The irradiated tissue is kept at a fixed, known distance from the source by spherical applicators to ensure a more uniform dose distribution. The tip of the electron drift tube sits precisely at the epicentre of a spherical plastic applicator, the size of which is chosen to fit the cavity after the tumour is excised, see Figure 1. Using this method, the walls of the tumour cavity are irradiated with a biologically effective dose (20 Gy to the tissue in contact with the applicator) that rapidly attenuates over a distance of a few centimetres. As a result, healthy tissue such as the vital organs is spared and the device can be used in an unmodified operating theatre as there is no need for lead shielding [27].

Another IORT approach is electron intraoperative therapy, pioneered at the European Institute of Oncology in Milan, Italy. In this technique, a portable linear accelerator is used to deliver a single dose of 21 Gy radiation during the surgery [28]. With this approach, it is necessary to perform the procedure in a specially shielded operating theatre for radiation safety considerations. The technique is currently being tested in a clinical trial, and results are eagerly awaited, as results from associated studies look promising [28, 29].

The safety and tolerability of the TARGIT technique has been established in a phase II study [30]. Starting in 1998, all 299 patients (with 300 cancers) who underwent breast conserving surgery for their breast cancer management received a single 20 Gy dose of radiotherapy during surgery. In these patients, this replaced 1 week of radiation to the tumour bed (boost radiation), and all patients received standard external beam radiotherapy to the whole-breast. A total of 32% of the patients were younger than 51 years; 57% of cancers were between 1 and 2 cm (21% >2 cm); 29% had a grade 3 tumour; 27% were node positive. The treatment was well tolerated by all patients, and with median followup of 60.5 months (range: 10–120 months), eight patients had developed ipsilateral recurrence: the 5-year Kaplan-Meier estimate for ipsilateral recurrence is 1.74% (standard error: 0.77). Based on the success of this study, a phase III superiority trial comparing TARGIT boost versus conventional boost will soon be launched.

In March 2000, an international, randomized controlled trial was launched comparing TARGIT versus whole breast external beam radiotherapy as a noninferiority study with the primary outcome of local recurrence. The original recruitment goal of 2232 (powered to test noninferiority; hazard ratio <1.25) was reached in early 2010, and the results were published [31]. 1113 patients were randomly allocated to TARGIT and 1119 to external beam radiotherapy. 14% of the TARGIT group also received external beam radiotherapy. At 4 years, there were six local recurrences in the TARGIT group and five in the external beam radiotherapy group. The Kaplan-Meier estimate of local recurrence in the conserved breast at 4 years was 1.20% (95% CI 0.53–2.71) after TARGIT compared with 0.95% (0.39–2.31) in the external beam radiotherapy group; the difference between the groups was not significant. The frequency of any complications and major toxicity was similar in the two groups, and radiotherapy toxicity was lower in the TARGIT group. The results of this study provide level-1 evidence that, for selected patients with early breast cancer, a single dose of radiotherapy delivered at the time of surgery using the TARGIT technique should be considered as an alternative to external beam radiotherapy delivered over several weeks.

Recruitment to the TARGIT Trial has been extended primarily to allow completion of subprotocols (quality of life, patient preference, health economics, and cosmesis). A pilot of the cosmesis subprotocol in 118 patients indicated a superior cosmetic outcome in the first year for those receiving TARGIT [32]. Results from a pilot patient preference study of 58 patients were used to determine if patients would accept the additional risk of 10-year recurrence in order to have TARGIT instead of conventional external beam radiotherapy. 54 (93%) of the subjects said they would undergo TARGIT if it offered equivalent or some added risk compared to EBRT [33].

Not all patients with early breast cancer were suitable for inclusion in the TARGIT Trial. In three major centres in the UK, Germany, and Australia, we offered IORT off-trial to a highly selected group of 80 patients with exceptional circumstances who could not receive standard external beam radiotherapy. Reasons for using TARGIT included 21 patients who had in-breast tumour recurrence in previously irradiated breasts, and 31 patients had clinical reasons such as systemic lupus erythematosus, motor neuron disease, Parkinson's disease, ankylosing spondylitis, morbid obesity, and cardiovascular or severe respiratory disease. 28 patients were included for compelling personal reasons, usually on compassionate grounds. After a median followup of 38 months, only two local recurrences were observed, which is an annual local recurrence rate of 0.75% (95% confidence interval, 0.09%–2.70%). These results indicate that TARGIT provides acceptable toxicity and good local control and offers an alternative to mastectomy in highly selected cases in whom conventional radiotherapy is not feasible or possible [34].

It has been found that wound fluid (taken from the drain over the first 24 h after surgery) stimulated proliferation, migration, and invasion of breast cancer cell lines; however, the stimulatory effect almost completely disappeared when fluids from TARGIT-treated patients were used. This was due to an alteration in the molecular composition and biological activity of the wound fluid and could provide some explanation for the very low recurrence rates found using TARGIT [35]. 

In summary, the evidence is mounting for TARGIT to replace whole-breast external beam radiotherapy for selected patients with early breast cancer. The technique is relatively easy to use, does not require shielding of the operating theatre, and largely protects healthy tissues. Furthermore, TARGIT is suitable for developing countries—an unusual example where a new health technology is more affordable than the existing standard and can optimise treatment and reduce the number of unnecessary mastectomies.

Looking forward, these encouraging results have prompted the initiation of new clinical trials using the TARGIT technique, for example, as the sole radiotherapy treatment in elderly women where long-term outcomes are less of a consideration; in nipple-sparing mastectomy to treat residual breast tissue behind the nipple-areolar complex; in cases of screen-detected DCIS with small focal lesions.

The treatment of breast cancer has undergone an evolution and is now poised for a revolution. New adjuvant hormonal therapies, novel chemotherapy, and targeted biological therapies are all helping to drive down mortality rates, and as this happens, cost effectiveness and patient acceptability become relatively more important. The radical approach of radiotherapy in early breast cancer is now being questioned. If IORT proves to be a suitable replacement for external beam radiotherapy, then many women will be spared several weeks of travelling back and forth to the radiotherapy centre. 

Furthermore, tens of thousands of women in the developing world who live hundreds of miles from a radiotherapy unit, or in countries that cannot afford the multimillion pound investment, will be able to enjoy the advantages of breast conservation instead of having to undergo mastectomy. 

The quest for the optimal treatment for early breast cancer has come a long way in the past 100 years or so. New technology, rigorously tested, will enable us to go a lot further. 

## Figures and Tables

**Figure 1:**
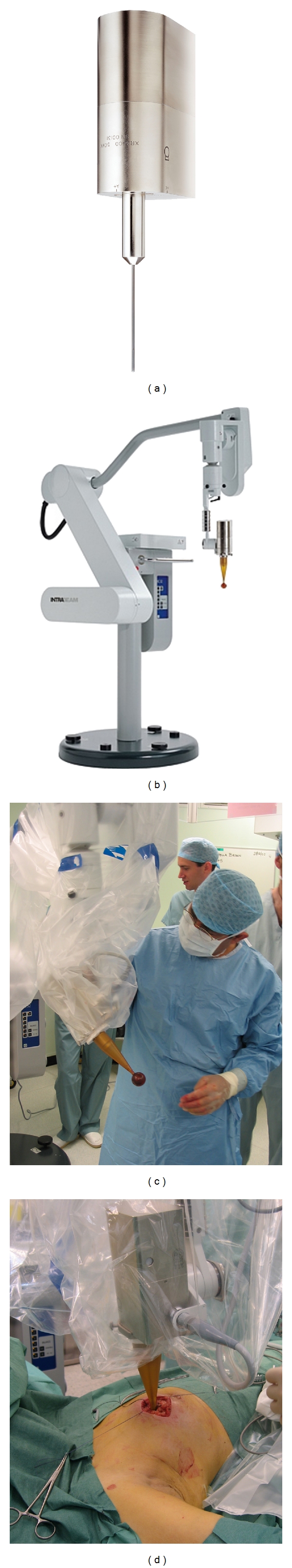
(a) The portable X-ray accelerator (Intrabeam). Soft X-rays are produced at the tip of the drift tube. (b) The intrabeam device mounted on the stand. The unit is portable and can be moved into the operating theatre as and when required. (c) Sterile drape and applicator in place, ready for positioning by the surgeon. (d) The applicator has been placed in the tumour bed and a purse-string suture is being applied to ensure conformity of the tissue to the applicator.

**Table 1:** Summary of evidence regarding the location of in-breast recurrences.

Year	Description	All LR	IBNP	True LR	Reference
2007	5318 patients with early breast cancer; all had lumpectomy and EBRT ± boost	91	17	81%	[36]
1993	567 women ± EBRT, 39 m median FUP. IBNP “>2 cm from primary site”	25	4	84%	[37]
1992	837 women ± EBRT, 43 m median FUP	131	19	85%	[38]
1992	488 women all with lumpectomy plus EBRT, 103 m mean FUP. “True” LR—“at or close to same quadrant as index case”	42	2	95%	[39]
1990	783 women, 80 m median FUP	91	17	81%	[40]
1990	381 women ± EBRT, 30 m FUP	15	2	87%	[41]
1990	496 women, 71 m median FUP	61	15	77%	[42]
1990	1593 women, 11 y median FUP	178	38	79%	[43]
1989	861 women, 5-6 years median FUP	93	19	80%	[44]
1984	231 women, 44 m median FUP	12	2	83%	[45]
1982	680 women	509	3	>99%	[46]
1982	28 women with DCIS treated by biopsy alone, FUP > 3 y	7	1	86%	[47]
1981	176 patients, median FUP 47 m	15	1	93%	[48]

LR = local recurrence (any recurrence in the ipsilateral breast).

IBNP = ipsilateral breast new primary (a LR that is some distance away from the site of the original tumour; precise definitions vary by study).

True LR = (All LR-IBNP)/(All LR) expressed as a percentage.
